# Digital Image Analysis of Vertebral Body S1 and Its Ossification Center in the Human Fetus

**DOI:** 10.3390/brainsci15010074

**Published:** 2025-01-15

**Authors:** Magdalena Grzonkowska, Katarzyna Bogacz, Andrzej Żytkowski, Monika Szkultecka-Dębek, Michał Kułakowski, Michał Janiak, Agnieszka Rogalska, Mariusz Baumgart

**Affiliations:** 1 Department of Normal Anatomy, The Ludwik Rydygier Collegium Medicum in Bydgoszcz, The Nicolaus Copernicus University in Toruń, 87-100 Toruń, Poland; mariusz.baumgart@cm.umk.pl; 2 Physiotherapy Department, Faculty of Physical Education and Physiotherapy, Opole University of Technology, 45-758 Opole, Poland; 3 Department of Anatomy, Faculty of Medicine, University of Social Sciences in Lodz, 90-113 Lodz, Poland; andrzej.zytkowski.anat@gmail.com; 4 Norbert Barlicki Memorial Teaching Hospital No. 1, Medical University of Lodz, 90-113 Lodz, Poland; 5 Faculty of Medicine, University of Social Sciences in Lodz, 90-113 Lodz, Poland; 6 Clinical Department of Orthopedics and Traumatology, Jan Biziel University Hospital nr 2 in Bydgoszcz, Nicolaus Copernicus University in Toruń, 87-100 Toruń, Poland; mkulakowski@poczta.fm (M.K.); michal.janiak@biziel.pl (M.J.); agnieszka.rogalska@biziel.pl (A.R.)

**Keywords:** fetus, ossification center, vertebral body

## Abstract

Objectives: The aim of the present study was to examine the growth dynamics of the first sacral vertebra and its ossification center in the human fetus, based on their linear, planar, and volumetric parameters. Methods: The examinations were carried out on 54 human fetuses of both sexes (26 males and 28 females) aged 18–30 weeks of gestation, which had been preserved in 10% neutral formalin solution. Using CT, digital image analysis software, 3D reconstruction, and statistical methods, the size of the first sacral vertebra and its ossification center was evaluated. Results: The first sacral vertebra and its ossification center grew proportionately according to fetal weeks. Conclusions: The numerical data obtained from computed tomography and the growth patterns of the body of the first sacral vertebra and its ossification center may serve as age-specific normative intervals relevant for gynecologists, obstetricians, pediatricians, and radiologists during fetal ultrasound screening. Our findings on the growth of the body of the first sacral vertebra and its ossification center may be useful in daily clinical practice, particularly in ultrasonic monitoring of normal fetal growth and in screening for congenital defects and skeletal dysplasias.

## 1. Introduction

Data regarding the timing of the ossification center’s appearance in individual vertebrae and their developmental patterns can provide critical insights into the maturity of the fetal skeletal system, one of the earliest and most rapidly developing systems during organogenesis. Moreover, early detection of spinal development abnormalities facilitates the identification of nervous system malformations and other fetal anomalies [1,2,3].

Each vertebra, except for the coccygeal vertebrae, has three ossification centers: one located in the vertebral body and one in each of the right and left neural arches. The first ossification centers emerge as early as the 8th week of fetal life in the neural arches of the upper cervical vertebrae, from which the ossification process progresses caudally. The ossification centers in the vertebral bodies begin to form in the 10th week of fetal life, initially in the lower thoracic vertebrae and the first lumbar vertebra. This process proceeds in both directions—more intensively toward the cranial direction and less intensively toward the caudal direction [3,4].

Abnormal spine length and curvature anomalies represent significant characteristics of various skeletal dysplasias (e.g., hemivertebrae, spina bifida), scoliosis, and segmental dysgenesis. These abnormalities may also be associated with genetic mutations, metabolic disorders, or exposure to teratogenic substances [5,6]. Fetal ultrasound biometry is currently considered the gold standard for assessing fetal growth. The most commonly used morphometric parameters include biparietal diameter, head circumference, and femur length. In addition, nomograms have been developed for other bone structures, such as the mandible, clavicle, scapula, vertebral arch, iliac bone, and foot length [5,6,7].

Measurement of the fetal sacral bone length is also recommended and should be included in routine fetal ultrasound assessment [8]. Incorrect determination of gestational age increases the risk of iatrogenic preterm births before the fetus reaches viability. Conversely, failure to accurately determine gestational age may result in intrauterine fetal demise in post-term pregnancies or complications related to prolonged gestation [5,6,7].

In addition to its role in estimating gestational age, the visualization of the S1 vertebra is crucial in diagnosing developmental abnormalities of the nervous system. It enables the assessment of the anatomical integrity of the lower spine and facilitates the identification of congenital defects, such as spina bifida, sacral agenesis, and sacrococcygeal teratoma [9].

In this study, we conducted an advanced morphometric analysis of the body of the first sacral vertebra and its ossification center in human fetuses. The objectives of the study were as follows:

- To quantitatively analyze the body of the first sacral vertebra and its ossification center with respect to their linear, planar, and volumetric parameters, aiming to establish reference values for specific gestational ages.
- To examine potential differences between sexes concerning the analyzed parameters.
- To calculate the growth dynamics of the analyzed parameters, including the identification of best-fitting mathematical models.

The conducted research in the field of translational neuroanatomy holds significant importance for understanding and diagnosing congenital nervous system defects and their impact on prenatal development. Precise analysis of the diameters of the first sacral vertebra (S1) and its ossification center may facilitate the early detection of abnormalities such as spina bifida or sacral agenesis, thereby enabling improved planning of perinatal care. The reference data obtained for the development of the S1 vertebra can serve as a valuable resource for studies investigating the structural foundations of developmental disorders in the lumbosacral junction.

## 2. Materials and Methods

### 2.1. Examined Sample

The study consisted of 54 human fetuses of both sexes (26 male and 28 female) aged between 18 and 30 weeks of gestation, obtained from spontaneous miscarriages and preterm births. They were collected and preserved in the collection of the Department of Normal Anatomy at Collegium Medicum in Bydgoszcz, Nicolaus Copernicus University in Toruń.

Morphometric studies were conducted from 1 January 2023 to 30 September 2023, at the Department of Anatomy of the Ludwik Rydygier Collegium Medicum of Nicolaus Copernicus University in Toruń. All fetuses included in the study were selected based on their unambiguous morphology and clinical documentation, with the requirement that they did not exhibit any obvious morphological defects or developmental abnormalities of the musculoskeletal system. As a result, fetuses with identifiable developmental diseases, such as congenital defects or intrauterine growth retardation, were excluded from the study. The gestational age of the fetuses was determined based on the crown–rump length and the known date of the mother’s last menstrual period. Tissue shrinkage resulting from formalin immersion had no impact on the obtained values [10,11,12]. Additionally, only those fetuses that demonstrated a strong correlation between the gestational age calculated based on crown–rump length and the age calculated based on the last menstrual period were included in the study. Table 1 presents the characteristics of the study group, including age, number, and sex of the fetuses examined.

### 2.2. Morphometric Measurements and Assessment of Ossification Centers

**Figure 1:**
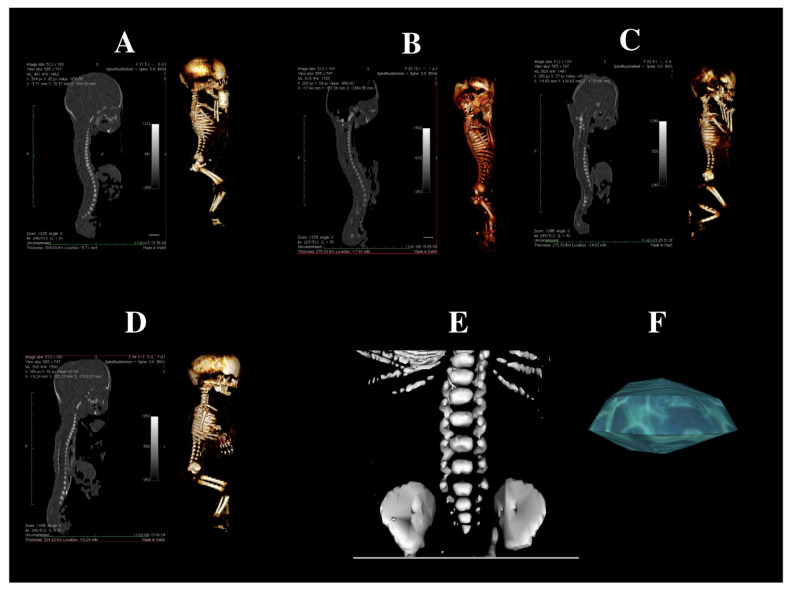
CT scans of a male fetus at 19 weeks (**A**), 21 weeks (**B**), 26 weeks (**C**), and 28 weeks (**D**) in sagittal projections, as well as 3D reconstruction in sagittal views, are presented. Additionally, a reconstruction of the lumbo–sacral vertebrae of a male fetus at 24 weeks in the frontal projection (**E**) is shown, including the ossification center of the S1 vertebrae (**F**) reconstructed using Osirix 3.9.

Using the Siemens Biograph 128 mCT scanner (Siemens Healthcare GmbH, Erlangen, Germany) located at the Department of Positron Emission Tomography and Molecular Imaging, Oncology Center, Ludwik Rydygier Collegium Medicum, Nicolaus Copernicus University in Bydgoszcz, Poland, fetal scans in DICOM format were acquired at 0.4 mm intervals (Figure 1).

The gray scale of the obtained CT images, expressed in Hounsfield units (HU), ranged from −275 to −134 for the minimum value, and from +1165 to +1558 for the maximum. Consequently, the window width (WW) ranged from 1.404 to 1.692, while the window level (WL) varied between +463 and +712. The imaging protocol was as follows: mAs—60, kV—80, pitch—0.35, FoV—180, and rotation time—0.5 s. The CT data specifics included a slice thickness of 0.4 mm, image increment of 0.6 mm, and kernel—B45 f-medium. Initially, the WW and WL parameters were automatically set by Osirix 3.9 MD software, and then adjusted by the observers to optimize the imaging of the studied structure.

It should be emphasized that the slice thickness of 0.4 mm may seem inadequate for such precise morphometric analysis. However, this slice thickness is the smallest available for the Siemens Biograph 128 mCT scanner and is bioethically acceptable for in utero measurements, as reflected in the protocol of the SpineRoutineTest (Child).

**Figure 2:**
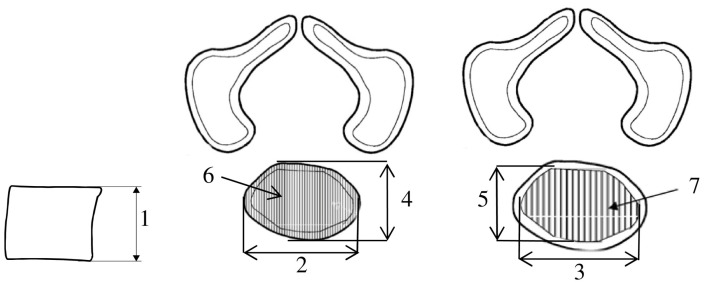
Measurement scheme of the body and ossification center of S1 vertebrae: 1—body height, 2—transverse diameter of vertebral body, 3—transverse diameter of body ossification center, 4—sagittal diameter of vertebral body, 5—sagittal diameter of body ossification center, 6—cross-sectional area of vertebral body, 7—cross-sectional area of body ossification center.

Measurements of the body of the first sacral vertebra and its ossification center were executed in a specific sequence (Figure 2).

Despite the cartilaginous stage of the ossification center of the sacral vertebra, its outlines were already clearly visible, allowing for volumetric assessment [13,14]. In each fetus, the assessment of linear parameters, projection surface area and volume of the body and ossification center of S1 vertebrae was carried out. Both the projection surface area and the volume of the body and ossification center of the S1 vertebra were semi-automatically calculated by outlining all regions of interest (ROIs) in the CT series, followed by the use of Osirix 3.9 MD to calculate the ROI volume.

The measurements for the body of the first sacral vertebra and its ossification center included:

1. Body height—the maximum distance between the superior and inferior edges of the vertebral body in the sagittal plane (Figure 2);
2. Transverse diameter of the vertebral body—the maximum distance between the lateral edges of the vertebral body in the transverse plane (Figure 2);
3. Transverse diameter of the body ossification center—the maximum distance between the lateral edges of the ossification center in the transverse plane (Figure 2);
4. Sagittal diameter of the vertebral body—the maximum distance between the anterior and posterior edges of the vertebral body in the sagittal plane (Figure 2);
5. Sagittal diameter of the body ossification center—the maximum distance between the anterior and posterior edges of the ossification center in the sagittal plane (Figure 2);
6. Cross-sectional area of the vertebral body—based on the determined contour of the vertebral body in the transverse plane (Figure 2);
7. Cross-sectional area of the body ossification center—based on the determined contour of the ossification center in the transverse plane (Figure 2).
8. Volume of the ossification center, calculated using advanced diagnostic imaging tools for 3D reconstruction, taking into account the position and the absorption of radiation by bone (Figure 1F).

### 2.3. Statistical Analysis

The numerical data obtained in this study were subjected to statistical analysis using Statistica 12.5 and PQStat 1.6.2 software. The distribution of the variables was tested using the Shapiro–Wilk test, and the homogeneity of variances was analyzed using Fisher’s test. The developmental dynamics of the analyzed parameters were characterized using linear and nonlinear regression analysis. The fit of the estimated curves to the measurement data was evaluated based on the coefficients of determination (R^2^). The relationship between the variables was also assessed using Pearson’s correlation coefficient (R). Differences were considered statistically significant at *p* < 0.05.

To continuously minimize the measurement and observational errors, all measurements were performed by a single researcher (M.B.) and verified by a second examiner (M.G.). Each measurement was taken three times under the same conditions but at different times (with one-day intervals) and then averaged. The inter-observer variation between repeated measurements was assessed using ANOVA analysis. The intraclass correlation coefficients (ICC) were statistically significant and showed excellent repeatability, as presented in Table 2.

## 3. Results

The mean values and standard deviations of the analyzed parameters for the body and ossification center of the S1 vertebra in human fetuses during the examined developmental period are presented in Table 3 and Table 4.

**Figure 3:**
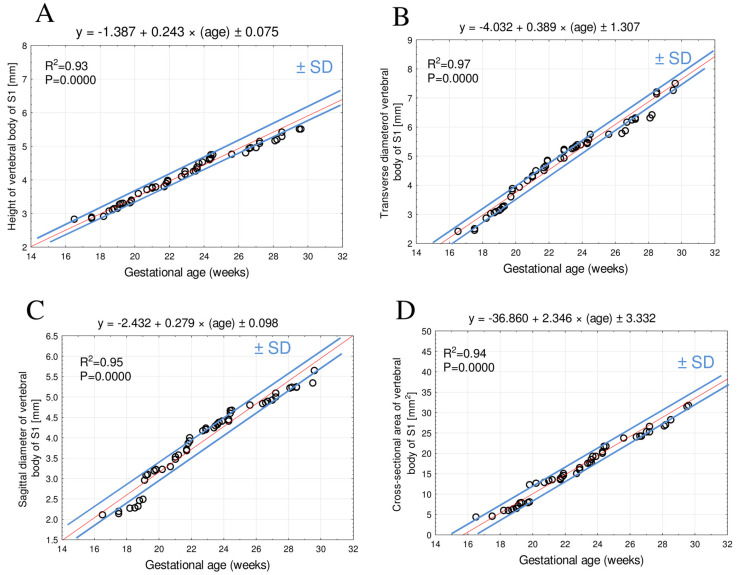
Regression lines for height (**A**) transverse diameter (**B**), sagittal diameter (**C**), and cross-sectional area (**D**) of the vertebral body of S1.

**Figure 4:**
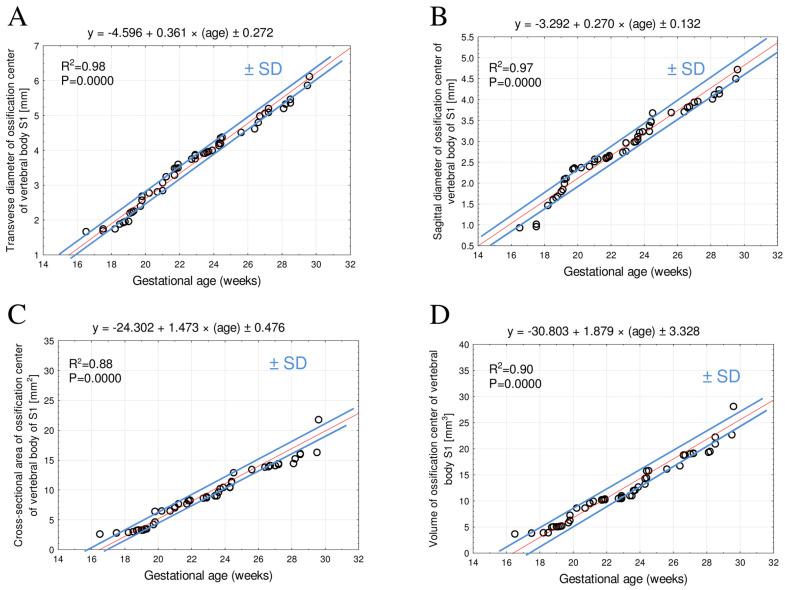
Regression lines for transverse diameter (**A**), sagittal diameter (**B**), cross-sectional area (**C**), and volume (**D**) of the ossification center of the S1 vertebral body S1.

For both the body and the ossification center of the S1 vertebra, no significant male–female differences were observed for any of the analyzed parameters (*p* > 0.05). Since the statistical analysis indicated no sex-related differences in the eight examined parameters of the body and ossification center of the S1 vertebra, numerical data for both sexes were aggregated, and a single growth curve was computed for each parameter. The growth patterns of the analyzed parameters are presented in Figure 3 and Figure 4.

### 3.1. Morphometric Parameters of the S1 Vertebral Body

At the gestational age range of 18–30 weeks, the mean height of the S1 vertebral body ranged from 2.89 ± 0.03 mm to 6.10 ± 0.92 mm. The growth pattern followed the linear function of gestational age: y = −1.387 + 0.243 × (age) ± 0.075, R^2^ = 0.93 (Figure 3A).

The mean transverse diameter of the S1 body between weeks 18 and 30 ranged from 2.61 ± 0.23 mm to 7.68 ± 0.51 mm. The growth pattern produced the linear function: y = −4.032 + 0.389 × (age) ± 1.307, R^2^ = 0.97 (Figure 3B).

The mean sagittal diameter of the S1 body between weeks 18 and 30 increased from 2.20 ± 0.07 mm to 5.78 ± 0.36 mm, modeling growth according to a linear function: y = −2.432 + 0.279 × (age) ± 0.098, R^2^ = 0.95 (Figure 3C).

The three linear diameters—height, transverse diameter, and sagittal diameter—exhibited a dynamic increase up to the 25th week of gestation, followed by a slight deceleration after the 26th week of gestation.

Finally, between weeks 18 and 30 the mean cross-sectional area of the S1 body grew from 5.08 ± 0.82 mm^2^ to 35.38 ± 7.22 mm^2^. Such an increase followed the linear function: y = −36.860 + 2.346 × (age) ± 3.332, R^2^ = 0.94 (Figure 3D).

### 3.2. Morphometric Parameters of the S1 Ossification Center

Between weeks 18 and 30, the mean transverse diameter of the S1 ossification center ranged from 1.73 ± 0.03 mm to 6.30 ± 0.39 mm, in accordance with the linear function: y = −4.596 + 0.361 × (age) ± 0.272, R^2^ = 0.98 (Figure 4A).

The mean sagittal diameter of the S1 ossification center at fetal ages of 18–30 weeks increased from 1.15 ± 0.27 mm to 4.85 ± 0.31 mm, following the linear function: y = −3.292 + 0.270 × (age) ± 0.132, R^2^ = 0.97 (Figure 4B).

In fetuses aged 18–30 weeks, the mean cross-sectional area of the S1 ossification center increased from 2.84 ± 0.06 mm^2^ to 23.28 ± 6.29 mm^2^, in accordance with the linear function: y = −24.302 + 1.473 × (age) ± 0.476, R^2^ = 0.88 (Figure 4C).

The mean volume of the S1 ossification center at fetal ages of 18–30 weeks ranged from 3.84 ± 0.05 mm^3^ to 29.88 ± 5.76 mm^3^, modeling growth according to the linear function: y = −30.803 + 1.879 × (age) ± 3.328, R^2^ = 0.90 (Figure 4D).

The cross-sectional area and volume exhibited a dynamic increase up to the 25th week of gestation, followed by a slight deceleration after the 26th week of gestation.

## 4. Discussion

Accurate determination of gestational age is crucial for preventing premature labor inductions and for appropriately timing procedures such as chorionic villus sampling, nuchal translucency assessment, amniocentesis, and planned cesarean sections [7]. The accuracy of gestational age prediction is highest during the first and second trimesters of pregnancy. However, as pregnancy advances, the reliability of measurements decreases due to the influence of various factors affecting all parameters in the third trimester. While no single fetal parameter can reliably estimate gestational age in the third trimester [15], nearly every significant clinical decision still depends on its accurate determination.

The fetal sacrum is routinely visualized during fetal spine ultrasound examinations. In contrast, the coccyx remains cartilaginous throughout the third trimester and at birth, undergoing ossification only postnatally. Consequently, the appearance of the coccyx differs from that of the other vertebrae, which have already undergone ossification. For this reason, the most distal ossification center in the spine is considered to represent the fifth sacral vertebra. Given that the fetal sacrum is an easily recognizable ossifying structure, its length can be measured via ultrasound in the sagittal plane [8,16,17].

Radiographic studies have demonstrated that the ossification process in the vertebral body occurs independently of that in the neural arches. Additionally, it has been suggested [17] that a significant correlation exists between crown–rump length and the number of primary ossification centers in the vertebrae. Consequently, it has been hypothesized that the number of vertebral ossification centers could serve as an indicator of fetal age [17]. A review of the literature revealed variability in the reported gestational age at which the ossification centers of the sacral bone become detectable via ultrasound [2,5,6,18,19,20,21,22] (Table 5). Previous studies have shown that ossification centers appear earlier in female fetuses [1,2,22]. However, Moradi et al. [21] did not observe a significant influence of fetal sex on the timing or pattern of ossification.

The first significant reports on the appearance of ossification centers were provided by Bareggi et al. [23], although they did not examine linear, planar, or volumetric parameters. In the S1 vertebra, they observed ossification centers at a crown–rump length (CRL) of 56 mm, corresponding to the 9th week of fetal life.

Diyva et al. [5] and Elkafrawy and Ahmed [7] suggest that the length of the sacral bone can serve as an important routine parameter, potentially aiding in situations where other standard measurement methods are either difficult to perform or lead to inaccurate estimations of gestational age. In our study of the body and ossification center of the S1 vertebra, we demonstrated that the examined parameters increased proportionally with gestational age. The coefficient of determination ranged from 0.88 to 0.98, indicating a very good fit of the growth model. Therefore, the measurement of a single sacral vertebra could also be utilized in cases where other assessments are uncertain or difficult to evaluate. It is important to note that this study is the first to examine, in detail and based on computed tomography imaging, the diameters of the body and ossification center of the S1 vertebra in relation to gestational age. In contrast, Szpinda et al. [11] were the first to examine the linear, planar, and volumetric dimensions of vertebral bodies using fetal material from human fetuses. However, the authors did not provide values for specific gestational weeks, instead reporting average values for the studied parameters, including the average height of the S1 vertebral body (3.71 ± 1.75 mm), the average transverse dimension (4.33 ± 2.37 mm), the average sagittal dimension (3.49 ± 1.86 mm), the average cross-sectional area (15.13 ± 10.97 mm^2^), and the average volume (71.88 ± 64.32 mm^3^).

Sherer et al. [18] were the first to define normal limits for sacral length and demonstrated a strong correlation between sacral length, gestational age, and other standard fetal growth measurements. In their study, they compared the sacral length in 506 normal fetuses and 80 fetuses with abnormal growth. Their findings revealed that the mean sacral length, measured in millimeters, was nearly equivalent to the gestational age in weeks. Moreover, they observed that the degree of linear dependence between gestational age and sacral length remained consistent throughout pregnancy, both in normal fetuses and those with growth abnormalities.

In the study by Elkafrawy and Ahmed [7], the average length of the sacral bone was found to be 18.17 mm at a gestational age of 16–24 weeks, 27.85 mm at 25–33 weeks, and 36.70 mm at 34–40 weeks. The authors demonstrated a proportional increase in sacral bone length as fetal age progressed.

Kędzia et al. [24] observed the most rapid growth in length (1.1 mm/week) and width (1.0 mm/week) of the S1 vertebra. In contrast, the slowest growth was noted for the length (0.37 mm/week) and width (0.64 mm/week) of the S5 vertebra. While all individual vertebrae exhibited steady growth in both analyzed diameters, the growth rate of the S1 was one and a half times faster than that of the S5 vertebra. No significant gender differences were observed in their study. This finding remains in line with our results, since we did not find any sex differences with respect to the body and the ossification center of the S1 vertebra in human fetuses.

Misra et al. [25] also demonstrated that sacral bone length increases linearly with advancing gestational age. Typically, the sacral bone length was found to be 1–3 mm shorter than the gestational age until around 30 weeks of pregnancy. After this period, the sacral bone length nearly equaled the gestational age, continuing this trend up to 40 weeks.

In contrast, Karabulut et al. [6] argued that sacral bone length does not change significantly during pregnancy, and that the correlation between gestational age and sacral bone length weakens in the third trimester. Their conclusions were further supported by similar data derived from anatomical autopsies of fetuses following abortion.

In our study, the examined group of fetuses ranged from 18 to 30 weeks of gestation, with only eight cases in the third trimester (from the 28th week) out of a total of 54 fetuses. The majority of our samples were from the second trimester, during which fetal spine development is characterized by dynamic and linear growth. In the subsequent weeks, a slowing of the growth rate and a plateau in the growth curves were observed, leading to weaker correlations, consistent with the findings of Karabulut et al. [6]. It is important to note that our sample consisted of only 54 fetuses, and fetal material is inherently unique, especially when analyzed using imaging techniques such as computed tomography (CT).

Additionally, our analysis was limited to the Caucasian population, which may restrict the generalizability of the results to other ethnic groups. Furthermore, the relatively small sample size may limit the detection of subtle growth changes and affect the precision of the conclusions regarding correlations, particularly for the smaller number of fetuses in the third trimester. Consequently, while our findings differ somewhat from those of Karabulut et al. [6], they also indicate a growth slowdown in the later stages of pregnancy, which may contribute to weaker correlations in the third trimester. Future studies with a larger sample size and a broader range of gestational ages may provide more accurate data for a comprehensive analysis of these growth patterns. It is important to highlight that our study’s morphometric analysis is distinctive due to its exclusive focus on the first sacral vertebra (S1), a significant deviation from the methodologies employed in previous studies [5,6,7,18,25]. This emphasis enabled a comprehensive examination of its structure, encompassing linear, planar, and volumetric parameters, through advanced CT reconstruction techniques. Such a detailed analysis may have been overlooked in studies that examined the entire sacral bone [5,6,7,18,25]. Consequently, our approach provides valuable insights into the development of the ossification center and vertebral body of S1 in human fetuses.

In this study, the selection of the first sacral vertebra (S1) is grounded in its critical role in diagnosing congenital spinal defects. As the first vertebra of the sacrum, the S1 vertebra is clearly visible on diagnostic images at an early stage of pregnancy (Table 5), making it a valuable reference point for assessing fetal spinal development.

The analysis of growth dynamics, which correlates a given parameter with fetal age for the body and ossification center of the S1 vertebra, may prove valuable in diagnosing fetal defects such as spina bifida, sacral agenesis, caudal regression syndrome, hemivertebra, butterfly vertebra, achondrogenesis, and osteogenesis imperfecta type II [2,26,27,28,29,30,31,32].

The use of computed tomography (CT) in spine imaging offers distinct advantages over traditional diagnostic methods, such as ultrasound. CT enables the acquisition of highly precise, three-dimensional images of bone structures, facilitating accurate measurements of dimensions and the assessment of vertebral shape. In contrast to ultrasound, which may have limited resolution when evaluating bone structures, CT provides superior accuracy in morphometric analysis. This is especially important in the early stages of pregnancy, when changes in spinal structure may be subtle and precise diagnosis is essential. Despite its advantages, the literature lacks detailed information on the quantitative analysis of the fetal skeletal system in specific weeks of pregnancy using CT, particularly when compared to ultrasound studies [33,34].

Computed tomography (CT) should be performed only when there are appropriate indications, using proper technical parameters and with consideration of the increased sensitivity of fetuses and infants to radiation compared to adults. While the benefits of low-dose CT for the fetus may outweigh the relatively small—yet still real—individual risks, CT should not be routinely used in clinical practice for diagnosing minor fetal bone abnormalities. However, it can be invaluable in clinically challenging cases where ultrasound diagnosis of severe or potentially life-threatening conditions remains inconclusive [33,34].

In our study, we applied mathematical models to describe the growth of the body and the ossification center of S1 vertebra. We observed that the height, transverse and sagittal diameters, the cross-sectional area of the body and ossification center, and the volume of the ossification center of S1 change proportionally with gestational age in weeks, indicating a steady increase between 18 and 30 weeks of gestation. It is particularly noteworthy that the volume of the ossification center of the vertebral body increased dynamically between the 18th and 24th week (Figure 4D), followed by a gradual slowdown in growth thereafter, as shown by the values in red bold in Table 4. The selection of the best-fit function is validated by the highest value of the coefficient of determination (R^2^), which accurately reflects the growth of the studied parameter and the degree of fit to the function.

In the study by Baumgart et al. [35] on the L4 vertebra, the authors demonstrated that in fetuses aged 18–30 weeks, linear diameters such as the height of the L4 vertebral body, the transverse and sagittal diameters of the vertebral body, and the ossification center grew logarithmically, while planar and volumetric diameters, such as the cross-sectional area and volume, grew linearly. In contrast, in our study, all parameters showed growth proportional to gestational age. This difference may be related to the more dynamic growth required for the L4 vertebra, which appears earlier and is larger in size compared to the S1 vertebra, thus influencing the growth patterns.

There is still limited information in the professional literature regarding the quantitative anatomy of the fetal skeletal system at specific gestational weeks using computed tomography (CT). Furthermore, no reports in the medical literature currently provide measurements of the vertebral body and its ossification center in the S1 vertebra of human fetuses, which limits the ability to conduct a comprehensive discussion on this topic. Consequently, the results of this study may contribute to a better understanding of fetal skeletal development, providing new numerical data relevant to its diagnostics and progression. Understanding the growth of individual vertebrae in human fetuses can benefit be useful in various fields, including anatomy, anthropology, forensic medicine, radiology, obstetrics, pediatrics, and orthopedics.

Our numerical data on the S1 vertebra may have significant implications for monitoring normal fetal growth and screening for congenital abnormalities.

### Limitations of the Study

The main limitation of the present study was the relatively narrow gestational age group, ranging from 18 to 30 weeks, and the small sample size (N = 54) of human fetuses.

## 5. Conclusions

1. No sex differences were observed for any of the morphometric parameters of the body and ossification center of the S1 vertebra.
2. The developmental dynamics of all studied parameters of the body and ossification center of the S1 vertebra demonstrated proportional growth with advancing gestational age, measured in weeks.
3. The obtained morphometric data on the body and ossification center of the S1 vertebra can serve as age-specific reference ranges that can support the estimation of gestational age and enhance in the ultrasonographic diagnosis of congenital abnormalities. Further research into the growth patterns and morphometric characteristics of the S1 vertebra’s body and ossification center is necessary to deepen our understanding of their development and potential clinical relevance.

## Figures and Tables

**Table 1:** Age, number and sex of the fetuses studied.

Gestational Age	Crown–Rump Length (mm)				Number of Fetuses	Sex	
Weeks (Hbd-Life)	Mean	SD	Min.	Max.		♂	♀
18	133.33	5.77	130.00	140.00	3	1	2
19	149.50	3.82	143.00	154.00	8	3	5
20	161.00	2.71	159.00	165.00	4	2	2
21	174.75	2.87	171.00	178.00	4	3	1
22	185.00	1.41	183.00	186.00	4	1	3
23	197.60	2.61	195.00	202.00	5	2	3
24	208.67	3.81	204.00	213.00	9	5	4
25	214.00		214.00	214.00	1	0	1
26	229.00	5.66	225.00	233.00	2	1	1
27	237.50	3.33	233.00	241.00	6	3	3
28	249.50	0.71	249.00	250.00	2	1	1
29	253.00	0.00	253.00	253.00	2	1	1
30	263.25	1.26	262.00	265.00	4	3	1
Total					54	26	28

**Table 2:** Intra-class correlation coefficients (ICC) values for inter-observer recurrence.

**Parameter of the Body of Vertebra S1**	**ICC**
Height	0.993 *
Transverse diameter	0.998 *
Sagittal diameter	0.996 *
Cross-sectional area	0.995 *
**Parameter of the Body Ossification Center of Vertebra S1**	
Transverse diameter	0.997 *
Sagittal diameter	0.996 *
Cross-sectional area	0.990 *
Volume	0.993 *

Inter-class correlation coefficients marked with * are statistically significant at *p*  <  0.0001.

**Table 3:** Height, transverse, and sagittal diameters and cross-sectional area of the body of vertebra S1.

Month	GA (Weeks)	N	Vertebral Body S1							
			Height (mm)		Transverse Diameter (mm)		Sagittal Diameter (mm)		Cross-Sectional Area (mm^2^)	
			Mean	SD	Mean	SD	Mean	SD	Mean	SD
V	18	3	2.89	0.03	2.61	0.23	2.20	0.07	5.08	0.82
	19	8	3.21	0.09	3.16	0.10	2.72	0.37	6.98	0.80
	20	4	3.42	0.12	3.81	0.15	3.22	0.02	10.25	2.60
VI	21	4	3.76	0.04	4.32	0.14	3.47	0.13	13.29	0.28
	22	5	3.89	0.08	4.69	0.15	3.84	0.13	14.26	0.61
	23	5	4.21	0.07	5.11	0.16	4.21	0.03	16.22	0.92
	24	9	4.52	0.16	5.42	0.10	4.43	0.13	19.61	1.51
VII	25	1	4.75		5.75		4.68		21.80	
	26	2	4.79	0.04	5.77	0.02	4.82	0.03	23.95	0.21
	27	5	5.02	0.09	6.18	0.18	4.96	0.09	25.12	0.99
	28	2	5.19	0.02	6.37	0.08	5.23	0.01	26.85	0.21
VIII	29	2	5.36	0.08	7.18	0.04	5.25	0.01	28.30	0.00
	30	4	6.10	0.92	7.68	0.51	5.78	0.36	35.38	7.22

**Table 4:** Transverse diameter, sagittal diameter, cross-sectional area, and volume of the ossification center of the body ossification of vertebra S1.

Month	GA (Weeks)	N	Ossification Center of Vertebral Body S1							
			Transverse Diameter (mm)		Sagittal Diameter (mm)		Cross-Sectional Area (mm^2^)		Volume (mm^3^)	
			Mean	SD	Mean	SD	Mean	SD	Mean	SD
V	18	3	1.73	0.03	1.15	0.27	2.84	0.06	3.84	0.05
	19	8	2.08	0.16	1.85	0.2	3.31	0.15	4.93	0.41
	20	4	2.61	0.16	2.35	0.02	5.42	1.18	6.98	1.23
VI	21	4	2.99	0.2	2.51	0.08	7.09	0.48	9.45	0.55
	22	5	3.47	0.11	2.62	0.03	8.02	0.29	10.23	0.1
	23	5	3.83	0.07	2.88	0.12	8.8	0.16	10.74	0.25
	24	9	4.07	0.15	3.24	0.17	10.27	0.78	13.03	1.5
VII	25	1	4.38		3.68		12.9		15.8	
	26	2	4.57	0.07	3.7	0.01	13.6	0.28	16.4	0.42
	27	5	5.03	0.15	3.9	0.07	14.16	0.19	18.98	0.18
	28	2	5.27	0.09	4.07	0.08	14.8	0.57	19.4	0.14
VIII	29	2	5.42	0.08	4.19	0.06	16.05	0.07	21.55	0.92
	30	4	6.3	0.39	4.85	0.31	23.28	6.29	29.88	5.76

**Table 5:** Variations in gestational age at which sacral bone ossification centers can be visualized using ultrasound.

The Observed Ossification Center of the Sacral Bone During the Ultrasound Examination	GA (Weeks)	Authors
S1–S5	14	Karabulut et al. [6]
S1–S5	15	Caughey et al. [20]
S1–S5	15	Tekani et al. [5]
S1-S5	16	Sherer et al. [18]
S1–S5	16	Ozat et al. [19]
S1–S2	15–17	Moradi et al. [21]
S1	15	De Biasio et al. [2]
S2	17	De Biasio et al. [2]
S3	17	Moradi et al. [21]
S4	20	Moradi et al. [21]

## Data Availability

The original contributions presented in this study are included in the article. Further inquiries can be directed to the corresponding author.
